# Innovative Multi-Target Estimating with Clutter-Suppression Technique for Pulsed Radar Systems

**DOI:** 10.3390/s20092446

**Published:** 2020-04-25

**Authors:** Jo-Yen Nieh, Yuan-Pin Cheng

**Affiliations:** 1 Department of Electrical and Electronic Engineering, Chung-Cheng Institute of Technology, National Defense University, Taoyuan 335, Taiwan; 2 Electronic Systems Research Division, National Chung-Shang Institute of Technology, Taoyuan 335, Taiwan; cyoungbeen@gmail.com

**Keywords:** linear frequency modulation, pulse compression, matched filter, Doppler shift compensation, Pulse-Doppler radar, moving target indication, comb filter, clutter suppression

## Abstract

Linear frequency modulation (LFM) waveforms have high Doppler-shift endurance because of the relative wide modulation bandwidth to the Doppler variation. The Doppler shift of the moving objects, nevertheless, constantly introduces obscure detection range offsets despite the exceptional Doppler tolerance in detection energy loss from LFM. An up-down-chirped LFM waveform is an efficient scheme to resolve the true target location and velocity by averaging the detection offset of two detection pairs from each single chirp LFM in opposite slopes. However, in multiple velocity-vary-target scenarios, without an efficient grouping scheme to find the detection pair of each moving target, the ambiguous detection results confine the applicability of precise target estimation by using these Doppler-tolerated waveforms. A succinct, three-multi-Doppler-shift-compensation (MDSC) scheme is applied to resolve the range and velocity of two moving objects by sorting the correct LFM detection pair of each target, even though the unresolvable scenarios of two close-by targets imply a fatal disability of detecting objects under a cluttered background. An innovative clutter-suppressed multi-Doppler-shift compensation (CS-MDSC) scheme is introduced in this research to compensate for the critical insufficient of resolving two overlapping objects with different velocities by solely MDSC. The CS-MDSC has been shown to successfully overcome this ambiguous scenario by integrating Doppler-selective moving target indication (MTI) filters to mitigate the distorting of near-zero-Doppler objects.

## 1. Introduction

During the targets searching stage, velocity and location information of the potential targets are unknown to radar systems. The unknown velocity can significantly degrade the target acquisition capability of the systems due to the Doppler shift distortion introduced by the object’s movement. Thus, a Doppler shift tolerated scheme is appreciative to deal with a variety of velocity object scenarios. In [1], a novel approach is proposed to integrated a linear frequency modulation pulse compression radar system with a time compression overlap-add technique to increase the signal-to-noise ratio. The transmitter divides a discrete linear frequency modulation chirp signal into overlapping segments and provides a significant processing gain. In [2], a new design of fast measurement to a linear frequency modulation is presented based on a linear amplitude comparison function that can ensure the accuracy of the measurement of multiple parameters. A study [3] verified the design of moving objects utilizing pulse compression technique and matched filter algorithm in linear frequency modulation in tracking the launch vehicle to follow the predetermined path or not. In [4], an 8 mm-range Gunn-diode oscillator was used in the experiment when the autodyne signal period duration was much longer than the delay time. The results of an autodyne short-range radar system with LFM in detecting moving reflecting objects were investigated. In [5], a new eigen-waveform design scheme was proposed to combine with the Range-Doppler map to identify moving targets where the detection performance was significantly improved over the wideband waveform and rectangular waveform. [6] announced a novel method to boost the detection probability of a radar system integrating eigen-waveform and pulse compression scheme. The hardware limitations were discussed under the scenarios of various waveforms. In [7], a new Doppler estimation method using space-variant synthetic aperture radar (SAR) imaging to enhance the performance of ship images was purposed and verified with GF-3 satellite SAR data. In [8], a new estimation method exploited moving target’s two-dimensional velocity parameters from SAR imaging for velocity compensation. The 2D motion parameters can be effectively computed by the matched compression. In [9], sea surface velocity estimation with the SAR technique is presented based on environmental satellite and an interferometric airborne SAR data-set.

In modern Pulse-Doppler radar systems, the coherent pulse train is commonly applied for power accumulation under the limitation of the maximum instantaneous power in the transmitters’ end. The ambiguity function |χ(τ,ν)| is widely used to exhibit the waveform characteristics in terms of the object time delay τ (location) vs. the Doppler frequency (f_d_) created by the velocity ν in the following section. In this paper, the ambiguity function of the LFM waveform is investigated and it shows a robust velocity tolerance of the LFM after the matched filtering (MF) in Section 2. The sidelobe level of LFM waveform after MF is high due to the rectangular modulation waveform. Nonlinear frequency modulation (NLFM) applies waveform smoothing techniques such as cosine spectrum shape [10] or Tayler windows [11] to mitigate the discontinuous transition region of the LFM. MLFM has advantages on lower side lobe level over LFM, but sacrifices wider main beam width and shaping power loss. However, this study focuses on the relationship between the target velocity and the detection range offset after MF of LFM and it is presented in Section 3. A novel MDSC method of sorting LFM detection pair out of multiple targets for overcoming the unknown range offset and its false estimation scenarios are shown in Section 4 and Section 5, respectively. Section 6 discusses an efficient Doppler frequency selective scheme, moving target indication (MTI), for pulse radar systems in order to overcome the MDSC’s disability in heavy clutter background scenarios. Section 7 presents case studies to an innovative CS-MDSC scheme and show how the MDSC can be improved and be functional under heavy clutter background scenarios. The comprehensive discussion is summarized in the conclusion section. 

## 2. Ambiguity Function of LFM Waveform

Linear frequency modulation (LFM) waveforms have wide modulation bandwidth compared with the relative narrow Doppler frequency (f_d__)_ variation of moving targets. It withstands Doppler interference by only sacrificing minimal energy loss in MF operation and it is also called pulse compression (PC) in the study. 

**Figure 1:**
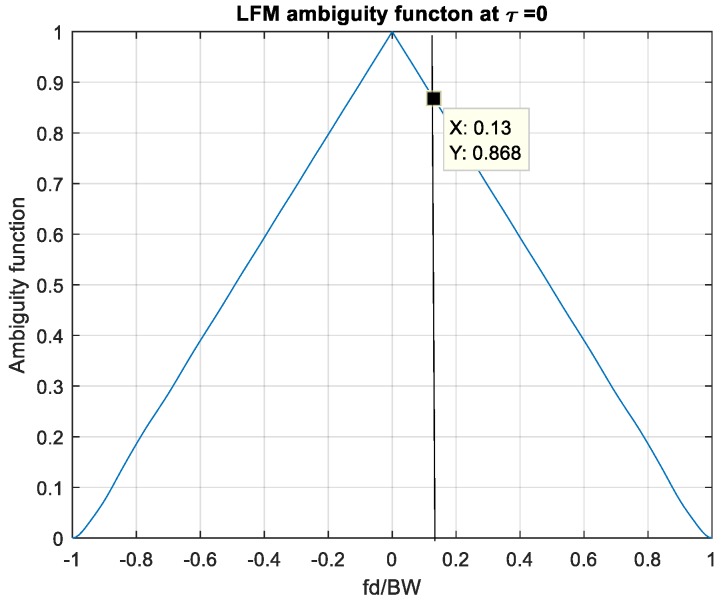
Normalized zero-time-delay ambiguity plot of linear frequency modulation (LFM) pulse. The vertical line marks the Doppler to bandwidth ratio of a moving target at f_d_/BW = 0.13.

Figure 1 shows the ambiguity plot at the zero-time delay of an LFM waveform with bandwidth = 1 MHz, pulse width = 1 s. In this chart, the frequency domain is normalized by the signal bandwidth for studying the relationship between the Doppler shift and the modulation bandwidth. The energy loss by the Doppler shift of the LFM wave is computed as follows

(1) Loss=|ν|/BW.

where ν is the Doppler frequency shift from the moving target. The sign is positive when the target is moving toward the observation point and the sign is negative otherwise.

The PC amplitude vs. f_d_ /BW is defined as follows [12]

(2) Amp=1−|ν|/BW.

The Doppler frequency f_d_ caused by a moving target from a stationary observation point is calculated by

(3) $$f_{d}=2\times fc\times Vel/\mathrm{speed}\;\mathrm{of}\;\mathrm{light}.$$

where the carrier frequency is *fc* and the object velocity is in the shorthand of *Vel*.

For covering object velocity up to 20 Mach, from Equations (1) and (2), the Doppler frequency is calculated as f_d_ = 136 kHz with the carrier frequency *fc*= 3GHz and the f_d_/BW = 0.136. The energy under this velocity is

(4) $$\mathrm{Amp}=10\times \log_{10}(0.868)=-0.6148(\mathrm{dB}).$$

Unlike single tone pulse train waveform, the amplitude coverage of LFM has a linear decay without a vicious variation. 

**Figure 2:**
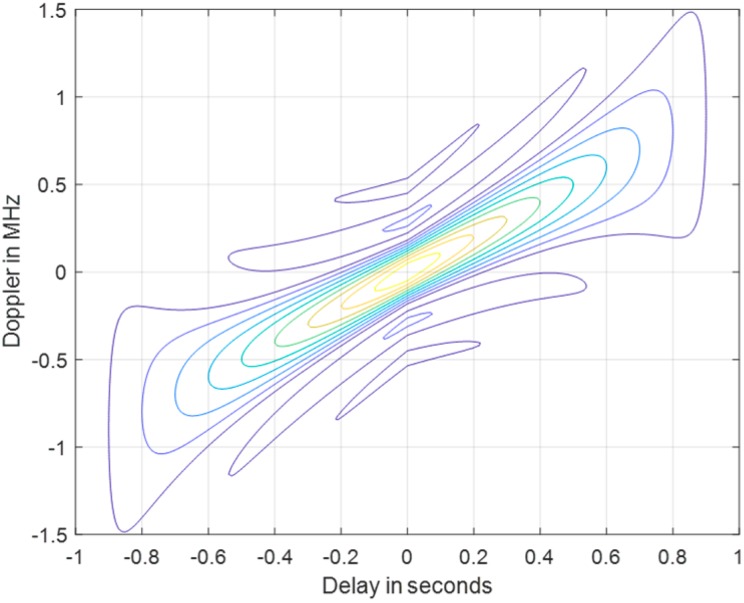
The contour plot of the LFM ambiguity function.

Despite the robustly Doppler-shift endurance of the LFM waveform, however, the time delay response also contains a linear offset along with the Doppler-frequency shift. The time delay offset can be observed in Figure 2, the contour plot of the LFM ambiguity function. The layout of the time delay diagonally corresponds to the Doppler shift. This offset results in the range error to Pulse-Doppler radars processing non-stationary moving target detection by matched filter detectors.

## 3. Range, Velocity Estimation by Offset of LFM Waveform

A single-chirped LFM waveform is defined as [12]

(5) $$x_{1}(t)=\mathrm{Rect}(\frac{t}{\tau_{0}})e^{j2\pi (f_{0}+\frac{\mu }{2}t^{2})}$$

where the pulse duration is *τ*_0_, the center frequency is *f*_0_, and the frequency chirping slope is *µ*. The sign of the slope *µ* indicates descending or ascending of the frequency increment. The steeper the slope, the more frequency difference in a fixed time period. 

**Figure 3:**
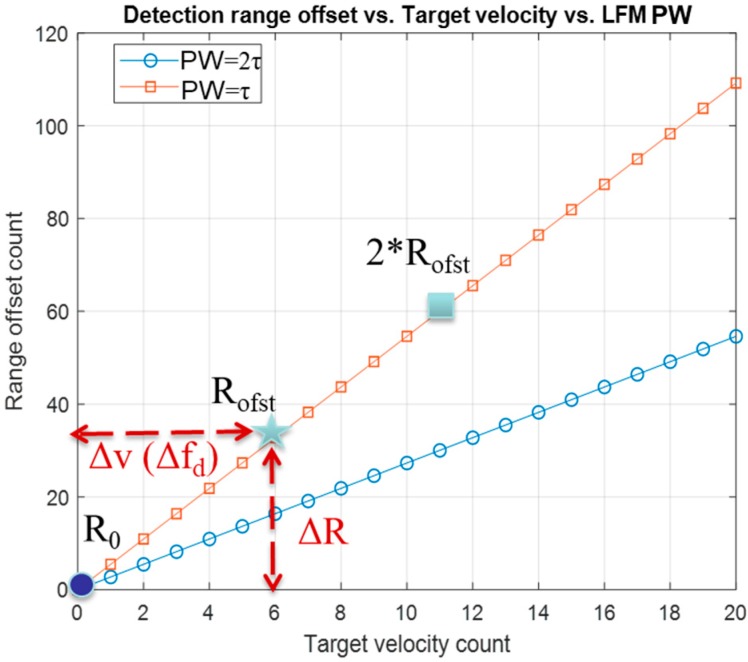
The linearity between target velocity and convolution range offset of a single chirped LFM waveform. The orange-squared line is the steeper chirping slope signal with pulse width (PW) = 2*τ* while the shows the flatter chirping slope signal with PW = *τ* in blue-circle line.

Figure 3 illustrates the phenomena of the range offset caused by the convolution of a reference simulated LFM waveform with a Doppler-shifted returning signal. The range offset is proportional to the amount of the Doppler shift (Δf_d_). 

The range offset of convolution is linearly proportional to the moving object Δf_d_ or Δν, which can be converted by Equation (3). The range offset changing rate is higher with the steeper chirping slope (pulse width (PW) = *τ*, orange-squared line) than the flatter chirping slope, PW = 2*τ*, LFM waveform (blue circle) under the same Δf_d_. Due to the linearity of a single chirp, the target velocity and detection range offset can be resolved from one another by the leaner ratio between f_d_/BW versus range offset R_oft_/µ. The equation is set up as follow [13]

(6) $$\frac{\left(\mathrm{PW}\times C/2\right)}{\mathrm{BW}}=\frac{R_{\mathrm{ofst}}}{\mathrm{fd}}$$

where the pulse width is PW, speed of light is C, bandwidth is BW, the detection range offset is R_ofst_ due to the Doppler shift f_d_. 

Derived from Equation (6)

(7) $$R_{\mathrm{ofst}}=\frac{\mathrm{fd}\times (\mathrm{PW}\times C/2)}{\mathrm{BW}}$$

R_ofst_ is proportional to f_d_ in a single chirped LFM waveform. With a stationary target, the detecting range offset is zero, which is shown as the blue dot in the origin in Figure 3.

However, in the target searching stage, without further target information, it is difficult to resolve the true moving target location by a single chirped LFM waveform. Therefore, a two chirped LFM scheme is introduced for resolving the true location of a non-stationary moving target.

Extended from Equation (5), a two chirped LFM waveform with two opposite slopes is derived as follows [14]

(8) $$\begin{array}{l} x_{2chirp}(t)\\ =\mathrm{Rect}(\frac{t-0.25\tau_{0}}{0.5\tau_{0}})e^{j2\pi [f_{0}(t-0.25\tau_{0})+\frac{\mu }{2}{\left(t-0.25\tau_{0}\right)}^{2}]}\\ +\mathrm{Rect}(\frac{t+0.25\tau_{0}}{0.5\tau_{0}})e^{j2\pi [f_{0}(t+0.25\tau_{0})+\frac{\mu }{2}{\left(t+0.25\tau_{0}\right)}^{2}]} \end{array}$$

where the waveform is composed of half of positive slope *µ* and a half of negative slope −*µ* LFM within a PW = *τ*_0_.

**Figure 4:**
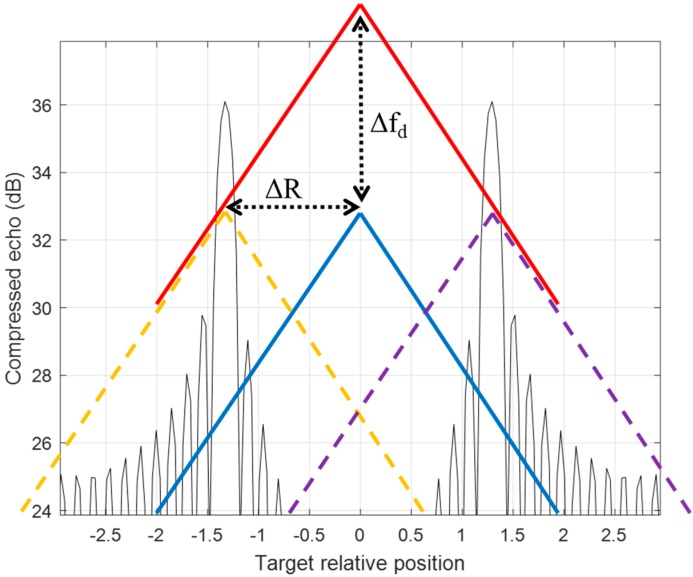
An up-down LFM waveform resolves two detection peaks from a non-stationary target by matched filter detectors.

In Figure 4, the example illustrates the phenomena of a detection pair of two equal range offset along with the true target position in the opposite direction after the matched filter detectors of a moving target with Δf_d_. When an up-down chirp referenced LFM signal (blue line) is shifted up by Δf_d_ (red line), the matched filter detector has two correlation points at ±ΔR locations offset. The up-chirp signal matches the reference signal at the yellow dotted line position on the left while the down-chirp one has a matched point at the purple dotted line on the right. With the detection pair of two chirps, the true location of the target, which is zero, can be resolved unambiguously by the mean of two locations of the detection pair [14].

Nevertheless, applying a two-chirped LFM waveform in multiple non-stationary targets scenarios, how to find the right pairs of the targets can be obscure without correct pairing information. There are three cases of ambiguous detection pair scenarios of two non-stationary moving targets.

**Figure 5:**
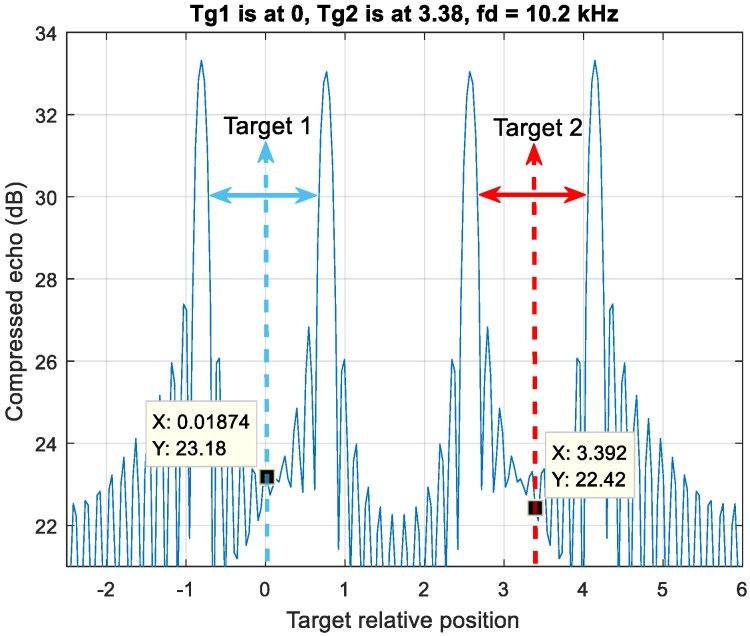
Case 1, an up-down chirp LFM waveform resolves four detection peaks by two moving targets. The detection pair of each target has no crossover.

Case 1 in Figure 5 demonstrates the detection pairs of two targets with the same velocity are right next to each other and the detection pairs are without crossover. Target one is at position zero, while target two is at position 3.3.

**Figure 6:**
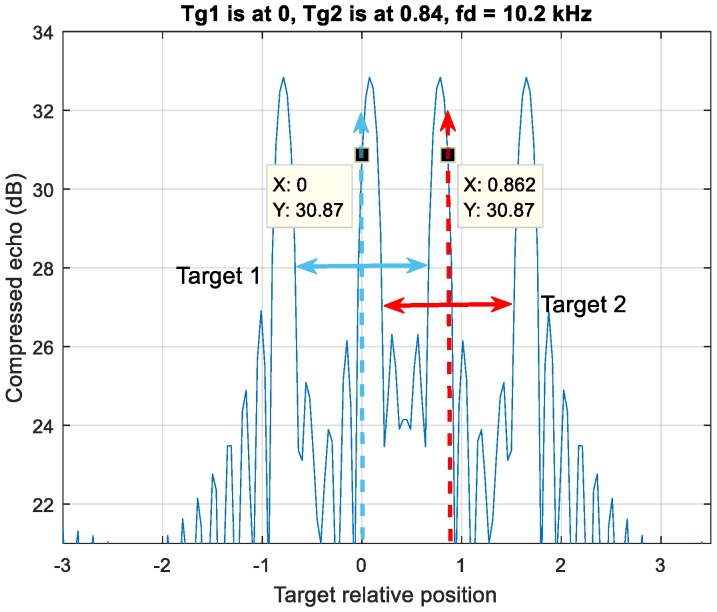
Case 2, an up-down chirp LFM waveform resolves four detection peaks by two moving targets. The detection pair of each target has one side crossover.

Case 2 in Figure 6 shows the detections of each target have one crossover with each other. Target one is at position zero, while target two is at position 0.86.

**Figure 7:**
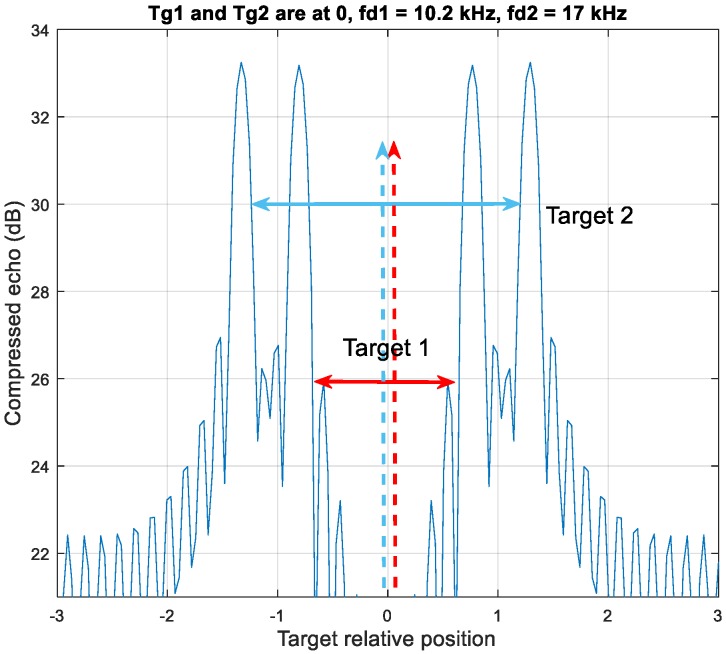
Case 3, an up-down chirp LFM waveform resolves four detection peaks by two moving targets. The detection pair of target 1 is enclosed by the pair of target 2.

In Case 3 in Figure 7, the target one pair is enclosed by the target two detection pairs. The two targets are overlapped at position zero.

Observing from the examples in Figure 5, Figure 6 and Figure 7, how to determine the right detection pair resolve the true location of each target is vague without the illustrative target-location marks. Thus, Doppler shift compensation (DSC) is applied to distinguish moving targets pair.

The DSC operation is computed as follows

(9) $$S_{\mathrm{offset}}(t)=S_{0}{\left(t\right)}^{*}\exp(j2\pi \times {\mathrm{fd}}_{\mathrm{offset}}\times t)$$

where the complex signal before DSC is S_0_(t), the DSC frequency step is f_d___offset_ I, and the signal after DSC is S_offse_t(t).

**Figure 8:**
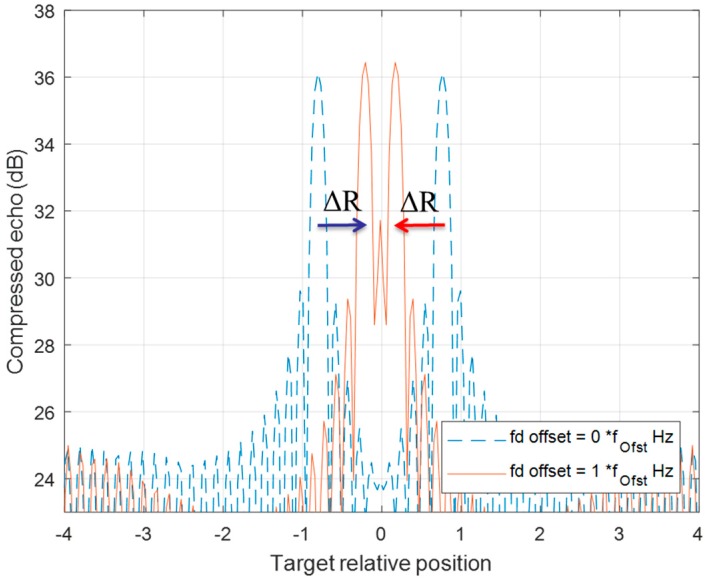
The range offset of a two-chirp detection pair of a moving target is processed by one fd_offset DSC. Two detections both have ΔR offset, but in the opposite direction.

**Figure 9:**
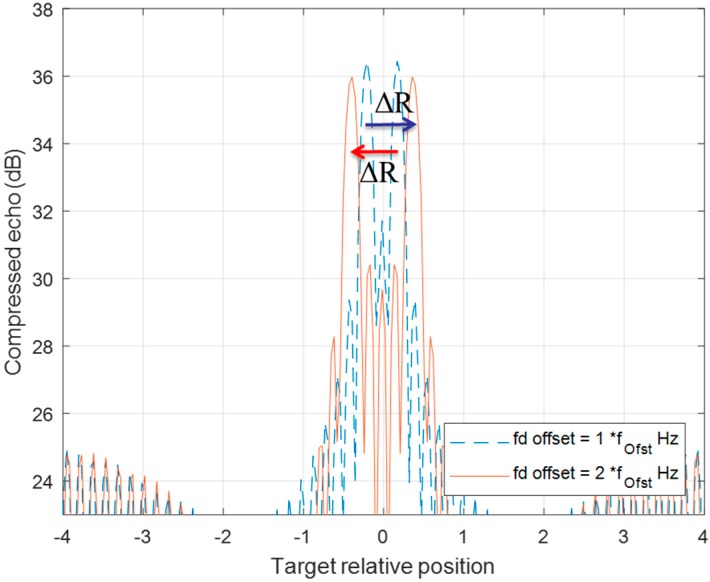
The range offset of a two-chirp detection pair of a moving target is processed by two f_d__offset DSC. Two detection lines crossover each other from one f_d__offset DSC (blue-dotted line) to two f_d__offset DSC (red line) with the same ΔR offset in opposite direction.

Figure 8 and Figure 9 demonstrate the behavior of a detection pair after a series of DSC operations. The detection of up-chirp LFM moves toward a positive direction by ΔR in each DSC, while the up-chirp LFM moves toward left each time with the same amount of ΔR. Even if the pair position has crossover in the Figure 9 scenario, the ΔR movement rule for each detection in this DSC operation is still valid.

## 4. Multiple-Doppler-Shift-Compensation (MDSC) Scheme for Sorting LFM Detection Pair of a Target

**Figure 10:**
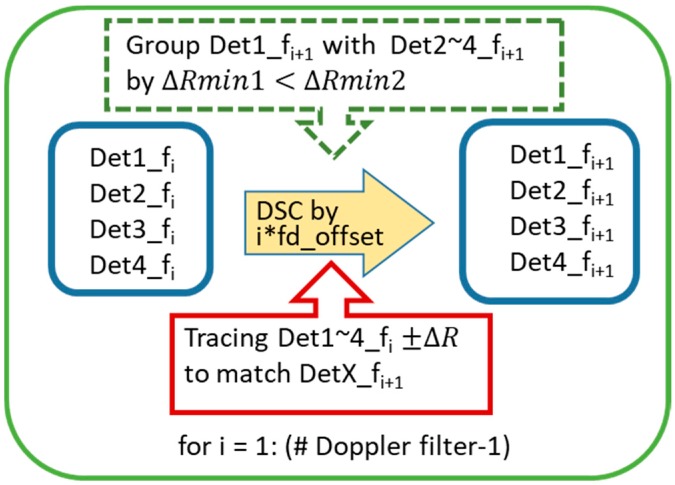
The processing flowchart of one Doppler filter in the multi-Doppler-shift-compensation (MDSC) scheme.

**Figure 11:**
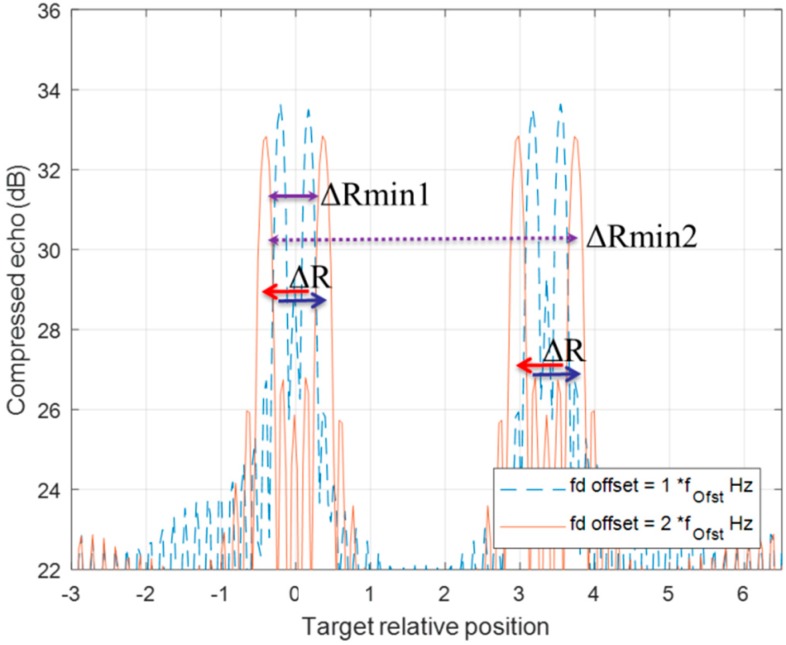
Two moving target MDSC of Figure 5 Case 1 scenario. The signal is traced from one f_d__offset Hz DSC (blue-dotted line) to two f_d__offset Hz DSC (red line).

Following the movement regulation of up-down chirp pair in DSC operations, a multiple-Doppler-shift-compensation (MDSC) scheme is applied to sort the detection pair out of multiple objects. In a two-moving-target scenario, one DSC operation flowchart is presented in Figure 10. There are five procession steps of one DSC operation:

Step 1.: An LFM signal containing two non-stationary targets after the correlation process yields four detection peaks located at Det1_fi to Det4_fi at the initial stage (left blue box) and an illustrative example is shown in Figure 11 as the blue-dash line.
Step 2.: An i-time f_d__offset DSC operation is applied to the two-target LFM signal, indicated as the yellow bold arrow in Figure 10.
Step 3.: Four detection peaks locate at Det1_fi_+1_ to Det4_fi_+1_ (right blue box) after the DSC operation, and a consequential example is shown in Figure 11 red line.
Step 4.: The red boxed arrow is the moving trend finding procedures, which associate Det1_fi to Det4_fi with Det1_fi_+1_ to Det4_fi_+1_ by finding the range offset matches the ±ΔR. Since the DSC operation only introduces a fix ΔR to each detection peak in the direction along with the LFM chirping slope. The ΔR directions after a DSC operation can be observed clearly in Figure 11. Those detection peaks with a positive ΔR offset are from up-chirped LFM, while the down-chirped ones introduce negative direction ΔR offsets.
Step 5.: Detection peak grouping (the green-dotted box) by finding: (1) these two peaks have opposite ΔR offset due to the up-down chirped LFM waveform being used; (2) the minimum distance between these presumed peaks. Figure 11 shows two possible detection pairs screened by rule (1), but the ΔRmin1 is accounted as the result in this DSC process because of the shortest distance selection assumption in the grouping procedure.

**Figure 12:**
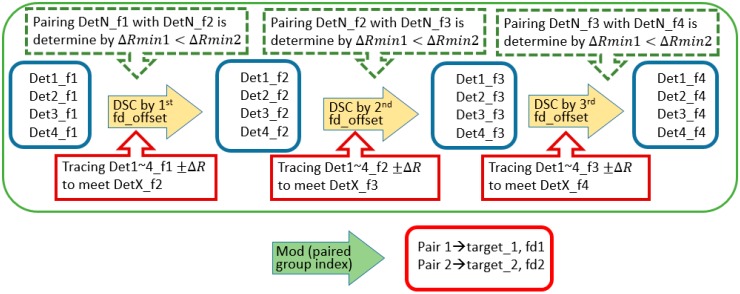
The decision flowchart of the multi-Doppler-shift-compensation (MDSC) scheme. A succinct three-Doppler-filter scenario is applied in this research.

The overall flowchart of three-MDSC is presented in Figure 12. There are three identical DSC operations with one to three times f_d__offset applied respectively to estimate the pairing indexes of two objects, as shown in the green dash-line box scheme. During each DSC operation, the location of DetX_f_i+1_ can only be found by a specific DetX_f_i_ ΔR, each DetX always has one possible match location at the next DSC (red box), and the grouping index is chosen by the condition of ΔRmin1 < ΔRmin2 (green-dotted box).

The three-MDSC operation yields three presumed detection pairs of these two targets from each DSC operation. The final target grouping pairs are determined by the majority grouping presumed results out of three DSC trials (green bold arrow). The true target location now can be resolved firmly by the mean of the detection pair, which is processed in the red box in the three-MDSC scheme.

The illustrative scenario of Case 1 in Figure 5 has three out of three trials correct detection peak estimation in three-MDSC operation.

**Figure 13:**
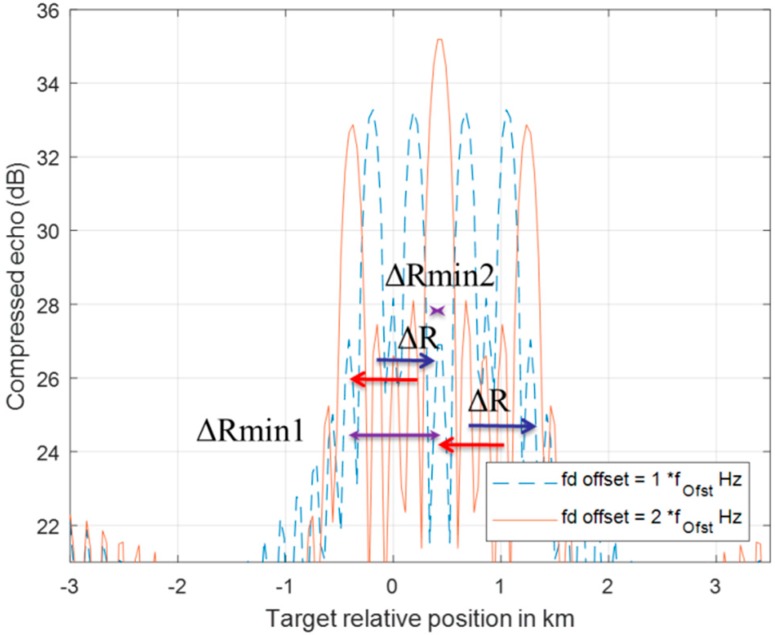
Two moving target MDSC of Figure 6 Case 2 scenario. The signal is traced from one f_d__offset Hz DSC (blue-dotted line) to two f_d__offset Hz DSC (red line). It is the special case, which the grouping rule finds a wrong pair out of three determining processes.

The Case 2 scenario in Figure 5, on the other hand, has one false pairing estimating result out of three DSC operations in three-MDSC when the detection pairs have crossover in a DSC shown in Figure 13. Nevertheless, the correct pairing index count is two out of three processes. The final decision is eventually correct (green arrow in Figure 10).

**Figure 14:**
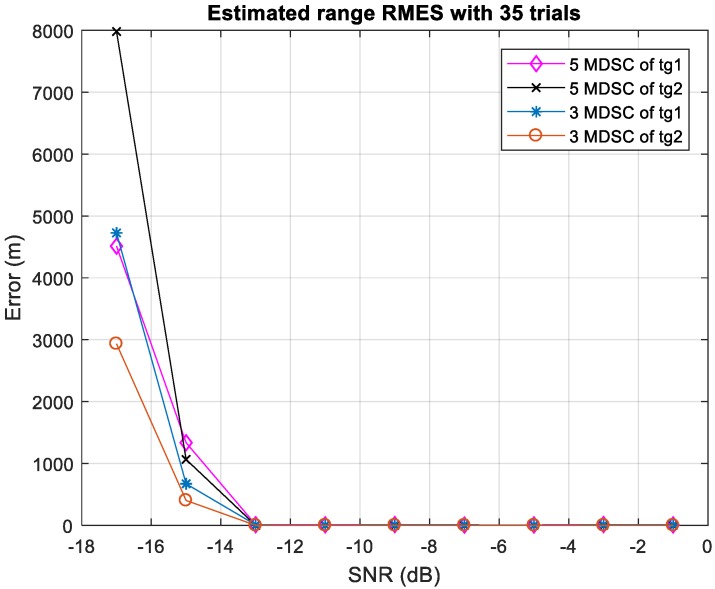
The 3-MDSC and 5-MDSC scheme evaluation error vs. SNR. The estimation error of target-one is marked as blue asterisk line and target-two is the red-circled line. The signal is with 30 dB matched filter pulse compression gain.

In prior research, five Doppler filters were used in the MDSC to resolve two target locations, respectively [14]. Since the pairing decision making is based on the majority grouping results of MDSC, a three-Doppler-filter MDSC yields three grouping pairs while the five-MDSC provides five grouping results. Both cases provide an odd number of pairing results, which means there is no ambiguity to make a majority decision out of the grouping results. Figure 14 shows the estimation accuracy comparison between three-MDSC and five-MDSC. Both MDSC schemes have accurate estimation results, as the SNR is above −15 dB while the PC ratio is 30 dB. As SNR < −15 dB, the detections are just too random for the MDSC scheme to have a correct matching pair to process, so the estimation cannot resolve targets’ location without corresponding information. The overall results prove that the succinct three-Doppler-filter MDSC has an equally-likely estimation capability as the original five-Doppler-filter MDSC scheme with less computational complexity by reduction in the DSC by two in each calculation cycle.

## 5. False Estimation Scenarios in MDSC

**Figure 15:**
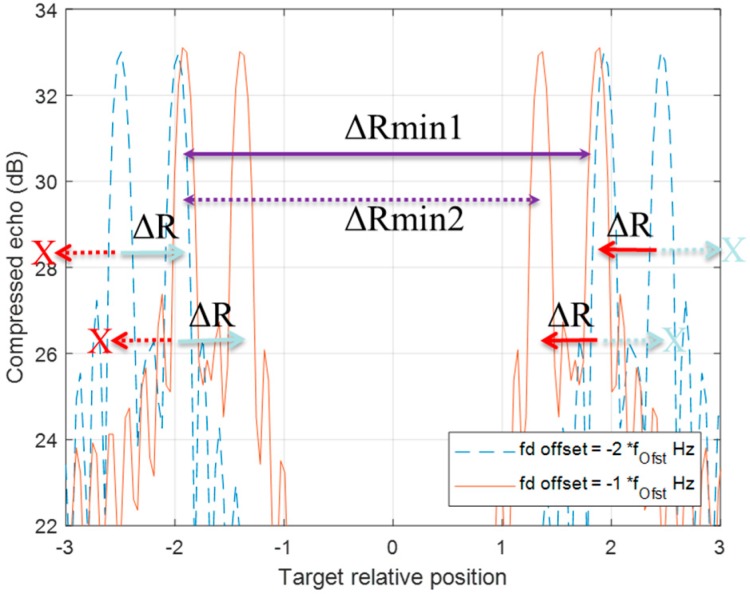
Two moving target MDSC of Figure 7 Case 3. The signal is traced by minus two f_d__offset Hz DSC (blue-dotted line) and minus one f_d__offset Hz DSC (red line). Since the target 1 pair is always enclosed by target 2 in such scenarios, the grouping rule always pairs the target 2 left detection with target 1 right detection at each DSC.

The MDSC scheme has a deficiency of correctly estimating the locations when the distance between two targets is smaller than the LFM range offset difference of two moving targets, as shown in Figure 15. In this case, the grouping results are never able to generate the correction pairing information.

**Figure 16:**
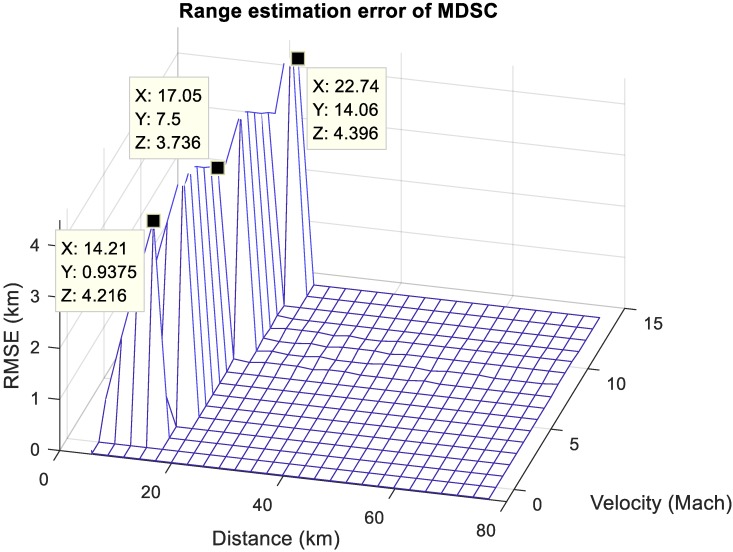
The range estimation error chart. Applying multi-Doppler-shift-compensation (MDSC) scheme estimates two non-stationary targets. The target distance is swept from 0 to 80 km while the velocity difference is swept from 0 to 15 Mach.

The ambiguous estimation scenarios of MDSC are grandly evaluated in Figure 16 with the targets velocity sweep from zero up to Mach 15 and distance is between zero and 80 km for a comprehensive analysis of unambiguous range estimation and possible false scenarios. The MDSC demonstrates a robust and accurate range estimation with zero root-mean-square error (RMSE) in unambiguous scenarios in Figure 5 and Figure 6. These two cases have a grand-coverage of most of the two-moving-target scenarios in terms of relative distances and velocities variation. However, the ambiguous Case 3 scenario in Figure 7 introduces the high RMSE in the range estimation due to the constantly miss-matching pairing results of two targets in MDSC, which is shown in the left of Figure 16. The Case 3 scenario indicates that the space between targets is insufficient to prevent one target detection pair to enclosure the other target detection pair, such as the distance (x label) < 22.74 km and the velocity difference = Mach 14.06 (y label) or distance = 14.2 km and the velocity difference > Mach 0.93. The higher the velocity difference, the longer the distance required to avoid these misleading scenarios [14].

These misleading results are introduced by the false pairing outcomes in MDSC processes in the Figure 7 Case 3 condition. These ambiguous conditions can be classified as two real-life scenarios:

(1) Two targets with a high-velocity difference are in a relatively close range. It resembles two high-velocity aircraft coming across each other at that specific detection timing. The estimation offset only results in a short-term discontinuity within a long-term detection trace which can be mitigated by simple smoothing and predicting schemes.
(2) A moving target is close by a near-stationary target, such as a target traveling through a cluster of low-velocity clutter. The slow-moving clutter creates a vast nearly-zero Doppler clutter background and gives MDSC a constant false estimating result under such conditions. These clutter distortion scenarios cause long duration and non-negligible misleading results for MDSC and need to be eliminated for a more practical application.

## 6. Low Velocity Target Suppression: Moving Target Indication (MTI)

To adapt the scheme to be practicable in the case (2) scenarios above, which resemble a crucially tactical scenario in which targets are blended into a heavy clutter background, integrating efficient clutter suppression schemes into MDSC is a substantial improvement to resolve this ambiguous scene. The techniques for implementing clutter filtering are the basis of the moving-target indication (MTI) scheme, which removes near-zero Doppler clutter spectrum and depth, and the width of the cut-off frequency of the filter is the factor of the number of pulses integrated and weighting coefficients of delay line applied on the pulse train.

Since this study is dealing with pulses transmitted at a pulse repetition frequency (PRF) f_r_, the received signal, from a given range, consists of one PRI = 1/f_r_ apart. The spectrum of such a signal is folded around f_r_/2 and centered around zero Doppler repeating every ±n*f_r_, n = 0, 1, 2… With this zero-Doppler canceling response, the nearly stationary clutters are the subject to be removed out of the non-stationary targets the periodicity in the filter response. The periodic frequency response of the filter resembles a comb; hence it is a so-called comb filter [15].

**Figure 17:**
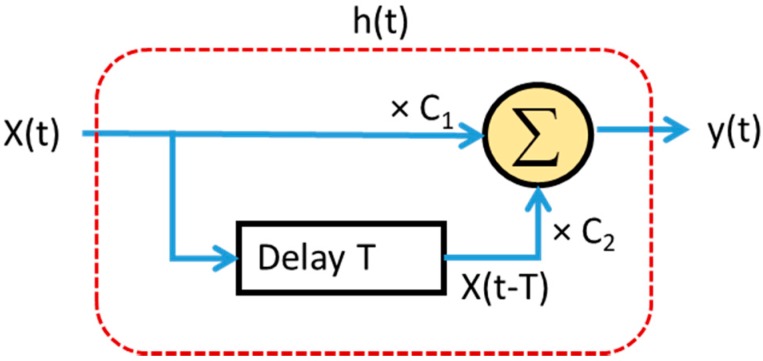
Single delay line canceler for two-pulse moving target indication (MTI).

The two-pulse MIT filter is also called a single delay line canceler which can be implemented as shown in Figure 17. It requires two distinct input pulse to yield out output. These sequential pulse trains are merged into a single pulse by feedback loops of the delay lines with specific coefficients weighing applying on each echo pulse. The output *y*(*t*) is defined as follows [16]

(10) $$y\left(t\right)=C_{1}x\left(t\right)+C_{2}x\left(t-T\right)$$

where the input *x*(*t*) is an n-pulse pulse train, delay *T* = PRI = (1/f_r_), and *C*_1_, *C*_2_ the weighting coefficients of each delay-line pulse summation operation.

The impulse response of the canceler is given by

(11) $$h\left(t\right)=C_{1}\delta (t)+C_{2}\delta (t-T)$$

**Figure 18:**
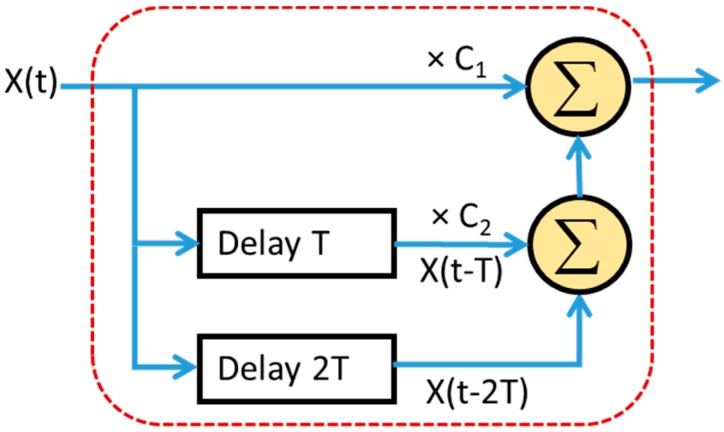
Double delay line canceler for three-pulse moving target indication (MTI).

The double-delay-line cancelers are shown in Figure 18 and it is also called the three-pulse MTI filter. There are three consecutive pulse train the impulse response is given by

(12) $$h\left(t\right)=C_{1}\delta (t)+C_{2}\delta (t-T)+C_{3}\delta (t-2T)$$

The output signal of a three-pulse input signal processed by a double-delay-line canceler is calculated as follows

(13) $$y\left(t\right)=C_{1}x\left(t\right)+C_{2}x\left(t-T\right)+C_{3}x\left(t-2T\right)$$

An MTI filter could be implemented using as little as two pulses and the filter high-pass response is determined by the number of pulses applied and weighting coefficients of the impulse response. Binomial coefficients are applied in the MIT filter frequency responses and a three-pulse delay line canceller is integrated with MDSC schemes to suppress the stationary object distortion to compensate for the ambiguous estimation in this study.

The binomial coefficients of multi-pulse MIT filters are given by

(14) $$C_{n}=\left(\begin{array}{c} n\\ k \end{array}\right)\times {\left(-1\right)}^{k}=\frac{n!}{k!(n-k)!}\times {\left(-1\right)}^{k},0\leq k\leq n,$$

where the number of pulse n and the sign of the coefficient is toggled by every *k*^th^ index.

**Figure 19:**
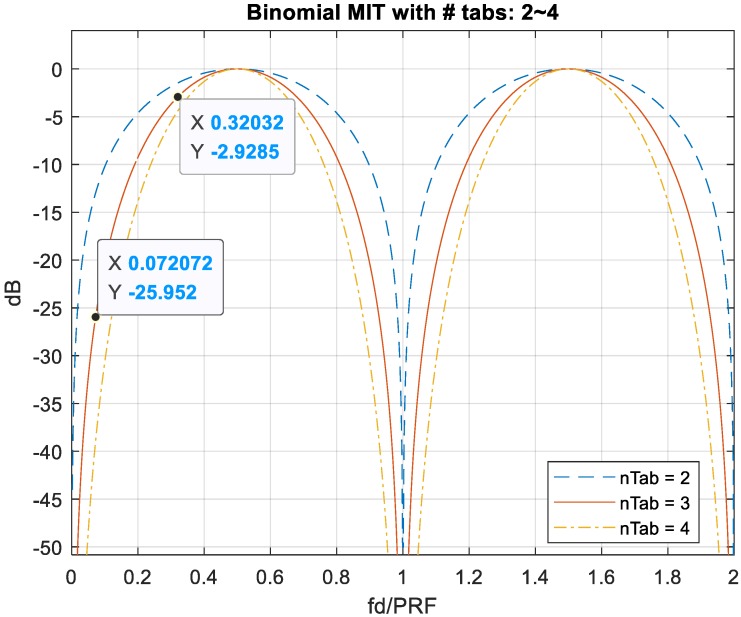
Frequency response of MTI comb filters applied binomial coefficients with the number of integrating pulses from two to four. The cut-off bandwidths are proportional to the number of delay lines applied.

The formula is also called Pascal’s rule or Pascal’s triangle [17]. The frequency response of the MIT filter with two to four pulses is shown in Figure 19. The zero-Doppler has a deep null point response repeated in multiples of f_r_ = PRF, by which the ambiguous low-velocity target factors are eliminated in the original MDSC. The cut-off bandwidth of near-zero-Doppler in MIT filter frequency response is proportional to the number of canceling line tabs, i.e., the more identical pulse trains integrated, the wider and deeper the cut-off bandwidth. Figure 19 shows the cut-off bandwidth of 2-tab MTI (blue-dash line) < 3-tab MTI (red line) < 4-tab MTI (yellow-dotted-dash line).

## 7. Two-Target, Clutter-Suppressed Multi-Doppler-Shift-Compensation (CS-MDSC)

**Figure 20:**
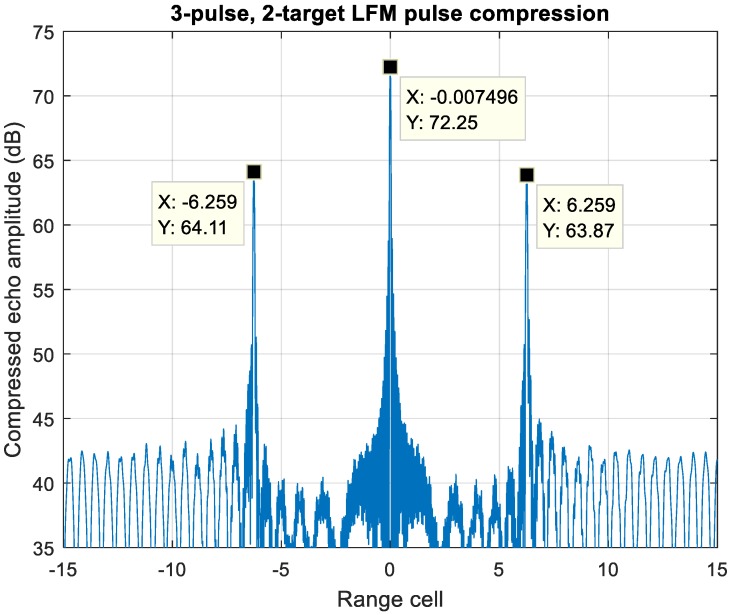
Pulse compressed echo of two overlapped targets. Target-one is zero Doppler, while target-two velocity is at Mach 15.

The ambiguous scenario with one near-stationary target has been discussed in Section 5 case (2). Figure 20 shows a typical unresolvable scenario to MDSC, which contains two targets at the same range cell with extremely velocity difference the target-one is stationary with zero-Doppler offset at the center, whereas the target-two velocity difference is at 15 Mach, which introduces a 102 kHz Doppler shift at S-band. The pulse compressed echo of up-down chirp LFM waveform has two peak values due to the Doppler shift at range cell ±6.25.

**Figure 21:**
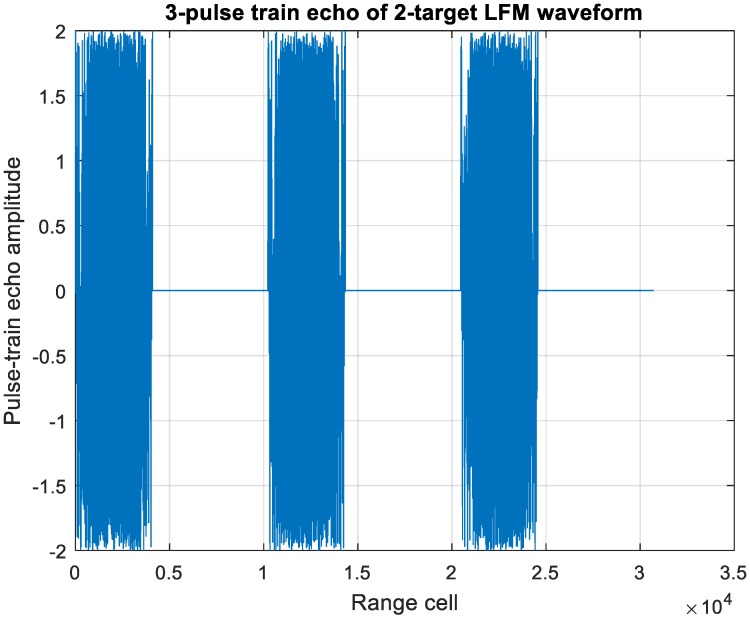
A 3-pulse LFM echo of two overlapped targets. Target-one is zero Doppler, while target-two velocity is at Mach 15.

For an MIT comb filter application, Figure 21 demonstrates a three-pulse LFM echo with two targets with such extreme location and velocity conditions described above with f_d_/PRF = 0 and 0.32, respectively. The frequency response of three-pulse MTI filter is shown in Figure 19; the target-1 echo with f_d_1/PRF = 0 gets suppressed significantly while that of target-2, f_d_2/PRF = 0.32, is in the passband of the filter.

**Figure 22:**
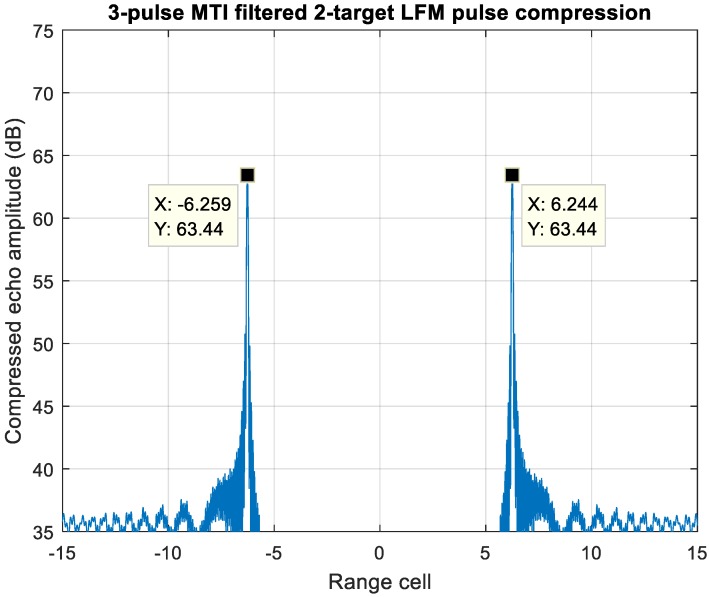
A 3-pulse MTI filtered echo of two overlapped targets. Target one is zero Doppler, while target two is at f_d_2/PRIL = 0.32.

Figure 22 displays the MTI processed echo of Figure 20, the zero-Doppler clutter is eliminated while the Mach 15 moving target echo remains, which leaves no ambiguity of the correctly pairing estimation.

**Figure 23:**
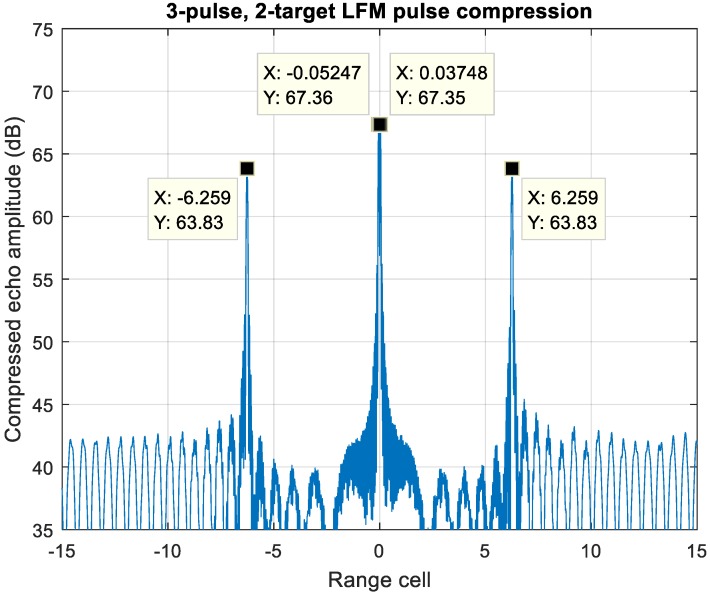
Pulse compressed echo of two overlapped targets. Target-one is Near-zero-Doppler, while target-two velocity is at 15 Mach.

Another case study of a slow-moving target overlapping with a high-velocity target, which is also an ambiguous scenario for MDSC, is shown in Figure 23. Both moving targets introduce a pair of detection peaks with range offset after PC of the LFM waveform. The slow-moving target resembles a near-zero-Doppler clutter, such as cloud or sea with the f_d_1/PRF = 0.072, while the second target resembles a high-velocity target, f_d_2/PRF = 0.32, travelling across this strong clutter background. Figure 19 shows that the near-zero-Doppler clutter also suffers significant attenuation at f_d_1/PRF = 0.072 for about 26 dB, whereas the second target with high velocity retains a strong response level at f_d_2/PRF = 0.32.

**Figure 24:**
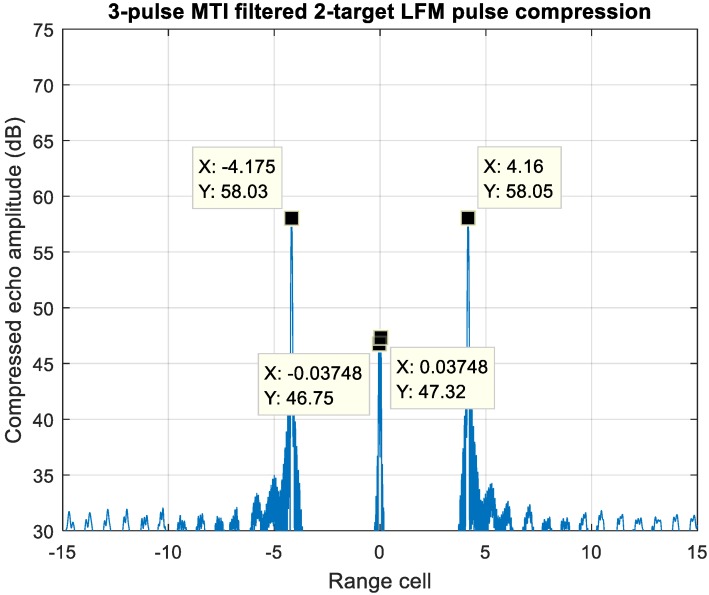
A 3-pulse MTI filtered echo of two overlapped targets. Target one is near-zero-Doppler, while the target-two velocity is at Mach 15.

Figure 24 shows the three-pulse MTI filtered echo of Figure 23. The −26 dB frequency response substantially drops down the target-one energy at f_d_1/PRF = 0.072. Since the outcome energy of target-one, which is implied as a low velocity clutter at range cell = ±0.037, has been significantly deteriorated and leaves 16 dB power difference between the detection pairs of two targets, this artifact signal can be removed completely by setting up a reasonable detection threshold, such as a constant false alarm rate threshold. Therefore, with the prior state of a properly designed clutter suppression scheme, the remaining detection pair at range cell ±6.2 can resolve the target-two range and velocity information unambiguously.

**Figure 25:**
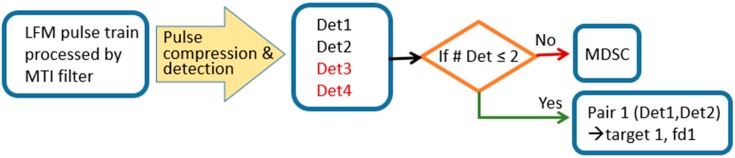
Clutter-suppression, multi-Doppler-shift-compensation (CS-MDSC) workflow.

To compensate the inapplicable scenarios of MDSC, a two-target, clutter suppression multi-Doppler-shift-compensation (CS-MDSC) workflow is illustrated in Figure 25. The process procedures begin with:

(1) An n-pulse LFM echo under a proper cut-off near-zero Doppler response MTI filter.
(2) Pulse compression and detection process.
(3) Number of detection count determination.
  - If the detection count is greater than two, then process MDSC to find the correct detection pair of LFM waveform for resolving these two targets’ locations and velocity.
  - If the number of detection is less than and equal to two due to the clutter suppression process, then there is no ambiguity on finding the right pair of the target. The location and velocity information of the moving target can be resolved by the remaining two or one detected signals.

## 8. Conclusions

The computational complexity and possible ambiguous estimation scenarios of two targets close by are the two noticeable drawbacks of the prior MDSC, which may create unwilling latency in the real-time system and false range/velocity estimation in inevitable heavy clutter background detection scenarios. There are two significant achievements in this study:

(1) Three-Doppler-offset MDSC operation has been proven as already providing sufficient range pairing information to have equally likely reliability as five-Doppler-offset MDSC, which was presented in the prior study. This succinct three-Doppler-offset MDSC workflow reduces the computational complexity by 40% as compared to five-Doppler-offset MDSC.
(2) The MTI comb filter clutter suppression scheme has been successfully integrated to prior MDSC by its pulsed Doppler periodic characteristics to eliminate the misleading pairing peaks from unwanted clutter-like signals. An innovative clutter-suppression multi-Doppler-shift-compensation (CS-MDSC) scheme has been introduced in this study and demonstrates the capability not only of maintaining the precise range and velocity estimation in most two moving targets scenarios as the original MDSC, but extract the moving target out of a clutter background, which is a magnificent improvement to adapt the MDSC scheme to broader and more realistic application scenarios.

Despite these advantages of CS-MDSC, further study of cognitive threshold selection algorithms and different coefficients applied on MIT comb filter should improve the scheme to adapt to more complex scenarios with more efficient processing algorithms. Also, the high sidelobe introduced by LFM rectangle waveform after MF leads out unresolvable detection pair of targets as the peaks are close. The shortage is more obvious, especially in dissimilar amplitude target scenarios. Thus, the future work is to look deep into the study of algorithm performance in presence of targets with dissimilar amplitudes and explore means to mitigate small target capture from larger target response.
